# Identification of resistant sources from *Glycine max* against soybean cyst nematode

**DOI:** 10.3389/fpls.2023.1143676

**Published:** 2023-03-07

**Authors:** Yun Lian, Ming Yuan, He Wei, Jinying Li, Binke Ding, Jinshe Wang, Weiguo Lu, Georg Koch

**Affiliations:** ^1^ Henan Academy of Crop Molecular Breeding, Henan Academy of Agricultural Sciences, Zhengzhou, China; ^2^ Qiqihar Branch of Heilongjiang Academy of Agricultural Sciences, Qiqihar, Heilongjiang, China; ^3^ College of Agricultural, State Key Laboratory for Conservation and Utilization of Subtropical Agro-Bioresources, Guangxi University, Nanning, China; ^4^ National Centre for Plant Breeding, Xinxiang, China

**Keywords:** soybean cyst nematode, *Heterodera glycines*, soybean, resistance, *Glycine max*

## Abstract

Soybean cyst nematode (SCN, *Heterodera glycines*, HG) is one of the severe pests in plant-parasitic nematodes, which impairs root development and causes severe losses in soybean production worldwide. Breeding SCN-resistant cultivars is an important measure for securing harvests without affecting the environment, as can be done with pesticides. The majority of genetic resources for plant pest resistances are found in wild or closely related species which are often difficult to use in breeding due to crossing barriers or close linkage with unfavorable agronomic traits. In this study, 12 soybean cultivars were evaluated for their marker haplotype at the *rhg1* and *Rhg4* SCN resistance loci and their SCN resistance tested against multiple races in environmentally controlled bioassays. The results showed that all 12 cultivars displayed Peking-type resistance marker haplotypes and all of them proved to be resistant to multiple SCN races. These cultivars provide potential for improving *H. glycines* resistance of soybean as donor parent in breeding and can contribute to reduce SCN field populations as part of a sustainable agriculture management.

## Introduction

Soybean (*Glycine max*) plays an important role in agricultural production (Hannah Ritchie and Roser, 2021), which is not only the source of vegetable protein and edible oil for human food but also the source of feed for animals and use in industrial processes. Plant pests and diseases are important factors limiting soybean yield, and the soybean cyst nematode (SCN, *Heterodera glycines*, HG) is one of the major plant-parasitic nematodes in soybean production. The yield losses caused by SCN in soybean production range from 10 to 30% on average each year (Koenning and Wrather, 2010; Bradley et al., 2021; Peng et al., 2021).

SCNs invade the soybean root as juveniles. The female larvae initiate in the root vascular cylinder, a so-called syncytium as a unique feeding site providing the nutrition for the nematode’s further growth and development (Klink et al., 2009). Through a feeding tube which is associated at the end of the nematode stylet and connected to the endoplasmic reticulum (ER) of the developing syncytium, the nematode secretes small molecules into the syncytium (Klink et al., 2007; Ali et al., 2013; Holbein et al., 2016). Subsequently, transcriptional and metabolic reprogramming invokes the compatible or incompatible interaction of the soybean host with the SCN. In incompatible interactions, the nematode’s development is arrested in the second-stage juvenile (J2) or third-stage juvenile (J3). In compatible interactions, the nematode can complete its life cycle within 4–5 weeks and turn into a solid cyst filled with hundreds of eggs after a male fertilized the female (Sobczak et al., 1999; Holbein et al., 2016; Mitchum, 2016).

Breeding soybean varieties with both high level of resistance and high yield offers an economic and ecological sustainable alternative to control SCN. In recent years, several reports from Missouri, USA, and the Huang-Huai Valley, China (Lian et al., 2016; Howland et al., 2018; Meinhardt et al., 2021) showed that formerly predominant SCN races shifted into more virulence races by cultivar-depending selection and that this evolution is expected to happen in many important soybean-growing areas. Even new virulent populations can develop like X12, which is a highly virulent SCN population found in Shanxi province, China (Lian et al., 2017; Lian et al., 2019b).

Although chemical and biological control agents including fungi and bacteria have been identified to be effective to manage this pest, environmental and economic concerns limit these approaches (Lin et al., 2022). Moreover, soybean is still being cultivated in small areas with low technology input or in fields with weak soil fertility by marginal farmers like in some parts of China. Thus, most of the management strategies for controlling SCN are seldom followed by such farmers or in soybean productions with low yield and revenue.

Therefore, it is desirable to identify resistance sources with broad-spectrum resistance to SCN and favorable agronomic traits, especially for yield traits, and utilize them to breed new resistant cultivars for long-term management of this devastating pest. Researchers tried to introduce strong SCN resistance sources such as PI 437654 and ZDD 2315 (Lian et al., 2017), but practical benefits from such germplasms were low due to the close linkage between resistance genes and adverse agronomic traits. So far, only very few accessions, like PI 88788 (around 90% of all resistant varieties are based on this resistance source), PI 209332, and Peking, were successfully used in the commercial soybean varieties for SCN resistance (Winter et al., 2006; Mitchum, 2016). However, more and more reports have shown that the sole use of resistances with single resistant genes such as PI 88788 is inefficient over time due to the development or selection of virulent nematode field populations (Niblack et al., 2008; Mitchum, 2016; Lin et al., 2022).

After years of research for the identification of SCN resistance resources with high agronomical value, a total of 12 soybean cultivars with multiple SCN resistances were identified in advanced breeding germplasms from China and in cultivars submitted for the national/provincial variety registration system in China. These cultivars have relatively high yield compared with Peking, Pickett, PI 88788, etc., and they are not derived from the single locus (*rhg1*) resistance source PI 88788. These results provide valuable genotypic and phenotypic information and methods for developing elite SCN resistant cultivars.

## Materials and methods

### Plant materials

12 SCN cultivars were previously identified as SCN resistant from larger collections of cultivar candidates or known cultivars. Zhonghuang 57, Handou 10, and Cangdou 11 derived from Huang-Huai Valley, China. Zhonghuang 57 and Handou 10 are known resistant cultivars with resistance to race 2 (Lian et al., 2019a; Wei et al., 2022). Cangdou 11 is screened by SCN bioassays with race 1 from a collection of 640 cultivar candidates. The other nine cultivars used in this study derived from Northeast China. Heinong 531 is a known resistant cultivar with resistance to race 3 (Wang et al., 2022). Heinong 84 is a cultivar showing *rgh1* and *Rgh4* resistance marker loci (Lian et al., 2022). Fengdou 1, Nenfeng 15, Nenfeng 17 (Chen et al., 2023), Nenfeng 18 (Chen et al., 2023), Nenfeng 19, Nongqingdou 24, and Qinong 5 derived from soybean breeder Dr. M. Yuan and were selected with race 3 in the field. The resistant and susceptible control cultivars Peking, PI 88788, PI 437654, and Lee were maintained by the soybean research group at the Henan Academy of Agricultural Sciences, Institute for Crops Molecular Breeding, Zhengzhou, China.

### SCN purification and inbreeding, propagation, and maintenance

Single SCN cysts were isolated from previously race tested soil samples (Lian et al., 2019b), which were selected for their potentially high uniformity to be infected with a single SCN race. Single cysts were used to inoculate susceptible Lee seedlings growing in soil sterilized for 2 h at 120°C. After four times of single-cyst propagation, SCN populations were considered as sufficiently inbred and race uniformity confirmed by race testing according to the SCN race model of Riggs (Riggs and Schmitt, 1988). Individual race populations of race 1, 2, 3, 4, and 5 were separately bulk multiplied several times, and cysts were stored in sterilized soil at 16°C in a dark room. The race populations were maintained in greenhouse culture pots on susceptible Lee plants, growing in soil from previous maintenance cultivation. SCN race 1, 2, 3, 4, and 5 are the most common races in the soybean production areas of China and worldwide (Lian et al., 2016).

### Nematode egg preparation

The adult SCN female cysts were washed off with pressure water from 30-day-old soybean roots placed on an 850-µm-pore sieve nested over a 250-µm-pore sieve. The eggs were released from inside the cysts using a rubber stopper on the surface of a 250-µm-pore sieve nested over a 210-µm-pore and an additional 25-µm-pore sieve (Faghihi and Ferris, 2000). Eventually, the eggs were collected on the 25-µm-pore sieve. Sucrose centrifugation with 20% sucrose/water solution was applied to separate the eggs from debris (Jenkins, 1964). The eggs were rinsed with water on a 25-µm-pore sieve to remove the sucrose solution and purified at 2,500 rpm. The eggs’ concentration was adjusted to 1,250 eggs/ml before use in experiments.

### Nematode resistance tests

The seeds used for resistance tests were germinated for 3 days in vermiculite at 25°C in the dark. The inoculation and infection of SCN to the soybean seedlings were carried out around 4 days after transferring single, germinated seedlings into cups (upper diameter × lower diameter × height: 70 mm × 50 mm × 90 mm) filled with sterilized soil. Lee was used as the SCN susceptible control. A total of 2,500 eggs were added with a membrane pump to each plant root by applying two times within 24 h 1,250 eggs with each 1-ml inoculation solution (eggs in water). Four weeks after infection, the roots were harvested and the cysts washed off as described above. The collected cysts were counted under an Olympus SZX16 stereomicroscope (Olympus, Japan). The average number of cysts per plant was calculated over five plants per soybean accession in each experiment and finally over three completely replicated experiments. A female index (FI) was calculated by dividing the average number of cysts on Lee (susceptible control) with the average number of cysts for the test accession (cultivar) multiplied by 100 for each experiment (replication), and finally, the overall average FI was calculated over the replications. According to Riggs and Schmitt (1988), a cultivar was designated resistant to SCN if the FI was ≤10%, whereas the cultivar was designated as moderate resistant, moderate susceptible, and susceptible to SCN with the FI values in the range of 10% < FI ≤ 30%, 30% < FI ≤ 60%, and FI > 60%, respectively.

### Molecular marker analysis of the SCN resistance loci *rhg1/Rhg4*

*rhg1* and *Rhg4* are the only two QTLs in soybean that have been consistently mapped in a variety of soybean germplasm sources so far, and the involved genes were identified and sequenced (Liu et al., 2012; Liu et al., 2017; Peng et al., 2021). These are also the two most prominent major resistance QTLs used in commercial germplasm (Peng et al., 2021; Lin et al., 2022). Kompetitive allele-specific PCR (KASP) markers for *rgh1* and *Rgh4*, developed by Shi et al. (2015), were applied to scan the presence/absence of these two major resistance QTLs in our cultivar collection. Of each cultivar, three plants were subjected to the marker analysis. Susceptible control cultivar Lee and the resistance control germplasms Peking, PI 88788, and PI437654 were used as marker control. Plants were genotyped with the two markers GSM381 and GSM383 for the *rhg1* locus on chromosome 18 and marker GSM191 for the *Rhg4* locus on chromosome 8 to determine the SCN resistance marker haplotype.

## Results

### Main agronomic traits of the 12 cultivars

The 12 cultivars were derived from Northeast China or the Huang-Huai Valley in Central China, the two major soybean-producing areas in China, and were registered as cultivars between 2004 and 2021 (**Table 1**). According to the official cultivar registration data, the cultivars Heinong 531, Fengdou 1, Nenfeng 15, Nenfeng 19, and Nongqingdou 24 are resistant to race 3; Zhonghuang 57 is displayed as SCN resistant but without any information about the test race; Nenfeng 15, Nenfeng 18, Nenfeng 19, Qinong 5, Handou 10, and Handou 11 do not have SCN testing results. The soybean yield of the 12 cultivars varied from 1,857 to 3,135 kg/ha in regional trials and from 1,942 to 2,911 kg/ha in prerelease trials. The oil content and protein content varied from 19.6 to 22.9% and from 37.8 to 42.6%, respectively. All the 12 cultivars are yellow seeded and have a higher 100-seed weight between 16 and 23.9 g as required for food use (**Table 1**). None of these registered varieties with SCN resistance have been well released in the local production area (Duan and Peng, 2014; Peng et al., 2021) except “Heinong 84”, which accounted for 4% of the soybean planting area in Heilongjiang in 2019 (Lian et al., 2022).

**Table 1:** Agronomic trait information according to the cultivar registration system, China.

Cultivar	Approval no.	Planting area	SCN testing/race	Regional trial years	Yield in regional trials (kg/ha)	Yield increase related to control (%)	Pre-release trial year	Yield in pre-release trials (kg/ha)	Yield increase related to control # (%)	100-seed weight (g)	Oil content (%)	Protein content (%)	Plant height (cm)	Seed coat color	Hilum color	Flower color	Pod fluff color
Nenfeng 15	Heishendou1994011	NE	race 3	1991-1993	1,857	5.5	1993	2,244	11.2	19.0	20.0	40.3	85	Yellow	Light brown	Purple	Grey
Nenfeng 17	HeiShendou2004012	NE	n.t.	2001-2002	1,881	5.6	2002	1,942	9.4	16.0	22.9	37.8	80	Yellow	Light brown	White	Grey
Nenfeng 18	HeiShendou2005009	NE	race 3	2001-2002	1,857	4.5	2003	2,195	10.1	21.0	22.7	38.2	90	Yellow	Light brown	White	Grey
Nenfeng 19	HeiShendou2006009	NE	race 3	2002-2003	2,040	6.5	2004	1,981	9.1	18.0	22.1	37.9	85	Yellow	Light brown	White	Grey
Fengdou 1	HeiShendou2006018	NE	race 3	2004-2005	2,025	17.6	2005	2,057	11.3	20.0	21.2	39.5	90	Yellow	Brown	White	Grey
Heinong 84	HeiShendou2017005	NE	n.t.	2014-2015	3,135	12.2	2016	2,891	13.0	22.0	19.6	40.8	100	Yellow	Yellow	Purple	Grey
Nongqingdou 24	Heishendou2018007	NE	race 3	2015-2016	2,467	9.1	2017	2,551	8.6	22.0	21.1	42.6	90	Yellow	Light brown	White	Grey
Qinong 5	HeiShendou2018006	NE	n.t.	2015-2016	2,615	11.2	2017	2,611	10.9	19.4	21.9	39.1	100	Yellow	Light brown	White	Grey
Heinong 531	HeiShendou20210004	NE	race 3	2019-2020	2,805	12.7	2020	2,752	13.6	21.3	22.3	38.2	85	Yellow	Yellow	White	Grey
Zhonghuang 57	GuoShendou2010005	HHV	race 3	2007-2008	2,876	3.1	2009	2,779	1.3	18.3	21.2	39.7	54	Yellow	Brown	Purple	Grey
Handou 10	Jishendou 2011002	HHV	n.t.	2009	2,760	–	2010	2,910	–	19.2	19.9	40.6	81	Yellow	Yellow	White	Brown
Cangdou 11	Jishendou 20190001	HHV	n.t.	2017	3,071	–	2018	2,911	–	23.8	20.1	41.3	99	Yellow	Yellow	White	Grey

#: NE, Northeast China; HHV, Huang-Huai Valley;

n.t.: not tested;

–: not reported;

Cultivars are sorted according planting area and first regional trial year.

### Evaluation of the SCN resistance using race 1 to 5

We evaluated the SCN resistance of the 12 cultivars in detail by testing with race 1, race 2, race 3, race 4, and race 5 in separate bioassays (**Table 2**). All 12 cultivars proved to be resistant to race 1, race 3, and race 5 except for Heinong 84 which was sensitive to race 5. All of the 12 identified cultivars showed various levels of resistance to race 2, i.e., one resistant cultivar, one moderately resistant cultivar, four moderately susceptible cultivars, and six susceptible cultivars. Related to race 4, only Zhonghuang 57 showed a clear resistance reaction, whereas two cultivars were moderately susceptible and the other nine cultivars proved to be susceptible against race 4. The combined race results showed that only Zhonghuang 57 proved resistant against all five races (resistance type like PI 437654), Heinong 531, Fengdou 1, Nenfeng 15, Nenfeng 19, and Nongqingdou 24 which turned out to be resistant to race 1, 3, and 5 (resistance type like Peking), and Heinong 84 resistant to race 1 and 3 (incomplete resistance type like Peking).

**Table 2:** The race testing results of the resistant elite cultivars/accessions to five soybean cyst nematode races.

	Cultivar/accession name	Race 1			Race 2			Race 3			Race 4			Race 5		
		Avg. no. of cysts per plant #1	Female index #2	Resistance level #3	Avg. no. of cysts per plant	Female index	Resistance level	Avg. no. of cysts per plant	Female index	Resistance level	Avg. no. of cysts per plant	Female index	Resistance level	Avg. no. of cysts per plant	Female index	Resistance level
*Susceptible control line*	Lee	157 ± 40.6 (110-209)	/	S	210 ± 32.8 (182-253)	/	S	98 ± 10.5 (87-108)	/	S	369 ± 100 (308-426)	/	S	401 ± 112 (112-487)	/	S
*Resistance check lines*	Peking	4 ± 1.3 (2-5)	2.4	R	171 ± 55 (114-246)	81.2	S	2 ± 0.8 (1-3)	2.0	R	239 ± 65 (201-275)	65	S	8 ± 2 (2-10)	2	R
	PI 88788	125 ± 45 (82-185)	79.9	S	45 ± 34.3 (6-89)	21.2	MR	5 ± 2.2 (3-8)	5.1	R	195 ± 53 (133-263)	53	MS	93 ± 49 (49-143)	23	MR
	PI 437654	1 ± 0.5 (1-2)	0.8	R	2 ± 1.8 (0-4)	1.0	R	1 ± 0.5 (1-2)	1.3	R	5 ± 1 (2-8)	1	R	4 ± 1 (1-5)	1	R
Resistant elite cultivars	Nenfeng 15	3 ± 1.3 (1-4)	1.6	R	169 ± 37.9 (127-204)	80.2	S	2 ± 0.8 (1-3)	2.0	R	219 ± 59 (71-334)	59	MS	10 ± 5 (5-15)	2	R
	Nenfeng 17	4 ± 1.7 (2-6)	2.2	R	76 ± 47.6 (37-141)	36.2	MS	4 ± 2.5 (1-6)	3.8	R	261 ± 71 (172-314)	71	S	3 ± 1 (1-4)	1	R
	Nenfeng 18	3 ± 0.6 (2-3)	1.6	R	111 ± 34.6 (72-147)	52.9	MS	7 ± 10.2 (1-22)	6.9	R	278 ± 75 (213-336)	75	S	7 ± 1 (1-8)	2	R
	Nenfeng 19	11 ± 11.1 (3-27)	6.9	R	145 ± 28.9 (108-177)	68.8	S	3 ± 1.7 (1-5)	2.6	R	244 ± 66 (125-375)	66	S	8 ± 5 (5-15)	2	R
	Fengdou 1	3 ± 1 (2-4)	2.1	R	97 ± 83.2 (13-171)	46.0	MS	4 ± 3.3 (1-8)	3.6	R	--	--	--	6 ± 3 (3-10)	1	R
	Heinong 84	4 ± 2.1 (1-6)	2.2	R	161 ± 23.3 (139-189)	76.7	S	2 ± 1.2 (1-3)	2.0	R	386 ± 105 (187-638)	105	S	329 ± 60 (60-383)	82	S
	Nongqingdou 24	3 ± 1.7 (1-5)	1.6	R	50 ± 35.8 (14-88)	23.9	MR	2 ± 0.6 (1-2)	1.5	R	201 ± 54 (99-296)	54	MS	7 ± 4 (4-11)	2	R
	Qinong 5	9 ± 4.7 (4-13)	5.4	R	135 ± 19.6 (114-158)	64.2	S	3 ± 1.5 (2-5)	3.4	R	277 ± 75 (163-363)	75	S	4 ± 1 (1-5)	1	R
	Heinong 531	4 ± 2.1 (1-6)	2.2	R	178 ± 43.9 (134-238)	84.8	S	2 ± 1 (1-3)	1.8	R	277 ± 75 (106-424)	75	S	6 ± 2 (2-9)	1	R
	Zhonghuang 57	5 ± 2.6 (1-7)	2.9	R	20 ± 12.5 (8-33)	9.5	R	2 ± 1 (1-3)	2.3	R	37 ± 10 (31-43)	10	R	6 ± 4 (4-11)	1	R
	Handou 10	2 ± 1.5 (1-4)	1.5	R	77 ± 42.1 (33-132)	36.4	MS	3 ± 2.6 (1-7)	3.3	R	367 ± 99 (356-381)	99	S	5 ± 3 (3-9)	1	R
	Cangdou 11	5 ± 1.7 (3-7)	3.0	R	145 ± 81.9 (56-231)	68.9	S	4 ± 3.6 (1-9)	3.8	R	303 ± 82 (181-443)	82	S	8 ± 2 (2-11)	2	R

#1: The average number of cysts per plant; ± standard deviation and minimum–maximum number of cysts per plant in parenthesis.

#2: The female index (FI) is calculated as the mean number of cysts of each line relative to the mean number of cysts per plant of the susceptible control line Lee68. Bioassay was replicated three times with five plants per race testing for each line.

#3: Resistance level definition: FI ≤10 = R; 10 < FI ≤ 30 = MR; 30 < FI ≤ 60 = MS; FI >60 = S.

–: no results.

Cultivars are sorted according planting area and first regional trial year.

### KASP molecular marker assay results

We performed KASP *rhg1* and *Rhg4* genotyping for the 12 cultivars and the control lines of the bioassay (**Table 3**). All of the 12 cultivars and the control lines Peking and PI 437654 carry the Peking-source marker haplotype for the *rhg1* and *Rhg4* resistance alleles (GSM381=G, GSM383=G, GSM191=G).

**Table 3:** Marker haplotypes at SCN loci *rhg1* and *Rhg4*, race testing phenotypes, and the corresponding resistance types of the 12 soybean cultivars and four known soybean reference lines.

	Cultivar accession name	Markers at locus *rhg1*		Marker at locus *Rhg4*	Resistance reaction to multiple SCN races					Resistance type according marker haplotypes at *rhg1* and *Rhg4*	Resistance type according to race testing
		GSM381 (Gm18: 645407) #1	GSM383 (Gm18: 1643660)	GSM191 (Gm08: 8361148)	Race1	Race2	Race3	Race4	Race5	Same haplotype as reference line	FI pattern comparable with reference line
*Susceptible control line*	Lee	T	C	C	S	S	S	S	S	Susceptible	Susceptible
*Resistance check lines*	Peking	G	G	G	R	S	R	S	R	Peking	Peking
	PI 88788	G	C	G	S	MR	R	MS	MR	PI 88788	PI 88788
	PI 437654	G	G	G	R	R	R	R	R	Peking	PI 437654
Resistant elite cultivars	Nenfeng 15	G	G	G	R	S	R	MS	R	Peking	Peking
	Nenfeng 17	G	G	G	R	MS	R	S	R	Peking	Peking
	Nenfeng 18	G	G	G	R	MS	R	S	R	Peking	Peking
	Nenfeng 19	G	G	G	R	S	R	S	R	Peking	Peking
	Fengdou 1	G	G	G	R	MS	R	--	R	Peking	Peking
	Heinong 84	G	G	G	R	S	R	S	S	Peking	(Peking)
	Nongqingdou 24	G	G	G	R	MR	R	MS	R	(Peking)	(Peking)
	Qinong 5	G	G	G	R	S	R	S	R	Peking	Peking
	Heinong 531	G	G	G	R	S	R	S	R	Peking	Peking
	Zhonghuang 57	G	G	G	R	R	R	R	R	(Peking)	PI 437654
	Handou 10	T	G	G	R	MS	R	S	R	Peking	Peking
	Cangdou 11	G	G	G	R	S	R	S	R	Peking	Peking

#1: Marker name and PCR according Shi et al. (2015). The number represents the genome coordinate based on the database: Gmax_275_v2.0.softmasked.

–: No results.

Cultivars are sorted according planting area and registration year.

## Discussion

In this study, 12 cultivars were genotyped for the known SCN resistance QTLs *rhg1* and *Rhg4* (Concibido et al., 2004; Cook et al., 2012; Liu et al., 2012; Liu et al., 2017). All 12 cultivars belong to the SCN resistance conferring the Peking-type marker haplotype and provide resistance against race 1, race 3, and race 5 (**Tables 2** , **3**) (Cook et al., 2012; Liu et al., 2012; Cook et al., 2014; Liu et al., 2017). Furthermore, in environmentally controlled SCN bioassays, the 12 cultivars were additionally phenotyped for resistance against SCN race 1–5 and all cultivars showed resistance against race 1, race 3, and race 5 except Heinong 84 which proved sensitive to race 5. All of the 12 cultivars have better yield compared with the control cultivars (1,942–2911 kg/ha in the prerelease trial). Although the yields of these SCN-resistant cultivars are already improved and competitive with the susceptible controls in registration trials in China, further yield and quality improvement can be facilitated using the molecular markers, as described in this study. Furthermore, the SCN bioassay results proved these cultivars as multiresistant, and there is a good chance that simultaneous resistances against race 1, race 3, and race 5 can be maintained during yield and quality breeding by applying the described molecular markers simplifying the selection and improving selection gain. Multiplex SCN resistance is also a valuable preventive measure against resistance breakdown and shift of virulence in the host populations (Meinhardt et al., 2021; Lin et al., 2022).

The special features of the 12 cultivars identified in this study are the multi-race SCN resistance and the relatively high yield, which are both simultaneously required for a sustainable cultivation of soybeans in the presence of SCN. Despite the yield advantage of the new SCN-resistant cultivars in the registration trials, only the cultivar Heinong 84 of the 12 cultivars was grown on 4% of Heilongjiang’s soybean planting area in 2019 whereas others have very little planting area as yet (Lian et al., 2022). Heinong 531 was just released in 2021 belonging to maturity group(MG I, Northeast China growing area) with 2,752 kg/ha in the 1-year prerelease trial and a yield increase of 12.0% compared with the local cultivars (Wang et al., 2022). Heinong 531 is the cultivar with the highest expectation of all the cultivars of MG I regarding SCN control and yield. In another study, Handou 10 showed an FI value of 28.0 and was described as MR (Wei et al., 2022). Here, Handou 10 achieved an FI of 36.4 which corresponds to an MS reaction. In general, the number of harvested cysts is environmentally affected by temperature, humidity, watering, etc., and even when the bioassay is carried out in an environmentally controlled climate room, there are minimal differences between different pots, experiments, and environments. It is not unexpected for a quantitative trait like FI that average values are varying to some degree. In this case, the overall conclusion is not much different regarding that MS and MR are intermediate scores which are neither resistant nor fully susceptible. Zhonghuang 57 also showed resistance to race 2 and race 4, two races that increasingly spread in soybean-growing areas worldwide (Xu et al., 2021; Lian et al., 2022). With resistances against all five races, Zhonghuang 57 is the most resistant cultivar in our study material and this multiplex resistance is comparable with one of the most resistant genetic resources for SCN resistance, PI 437654 (Shi et al., 2015). This result also shows the limitation of our molecular markers which are not able to differentiate between Peking-type resistance and PI 437654-type resistance. Zhonghuang 57 had a relatively good yield with 2,779 kg/ha in the prerelease trial in 2009 and a yield increase of 1.3% when compared with Huang-Huai Valley local cultivars (MG III). By using marker-assisted selection with the described KASP markers, this cultivar with that excellent SCN resistance profile is a valid candidate for further yield and quality increase to achieve market competitiveness for MG III cultivars and the Huang-Huai Valley production area.

Of the 12 SCN-resistant varieties described in this study, Heinong 84 was bred by combining molecular marker-assisted selection with traditional pedigree breeding, whereas the other varieties were only selected through traditional pedigree breeding. In the cultivar registration system, it is shown that each one of the parents of Nongqingdou 24, Fengdou 1, and Zhonghuang57 was resistant against SCN. There is no further information about the parents for these cultivars. Previous SCN resistance screening studies were often carried out with only one race like race 3 with no specific virulence, whereas in this study, the most common reported races in the soybean production area worldwide, race 1 to race 5, were included. In contrast to most resistance screening studies, the 12 soybean cultivars included here are proven of agronomic value with competitive yield, oil and protein content, and seed coat color, which makes them more easily accessible for modern breeding than other genetic resources like wild soybean species or minimally adapted landraces. Moreover, with Zhonghuang 57 we identified a cultivar with broad resistance against all five races which goes beyond the popular resistance sources like PI 88788 (*rgh1-b*) and Peking (*rgh1-a*, *Rgh4*). Zhonghuang 57 can be used in areas where race 2 or race 4 are of major yield concern. Nematode populations can overcome the resistance of a single resistance locus within a decade like it was described for PI 88788, the most known and used SCN-resistant source (Niblack et al., 2008; Howland et al., 2018; Meinhardt et al., 2021). Unique resistance types that are different from PI 88788 and best, based on multiple resistance loci like Zhonghuang 57, will be valuable for developing soybean cultivars with potentially durable resistance to SCN.

Cultivars with the same genetic sources of resistance do not necessarily have the same levels of resistance in the field (Niblack et al., 2002). Therefore, before using one of these varieties as resistance donor in a new breeding program, it is advised to confirm the SCN resistance performance in infested fields. Marker-assisted selection (MAS) of the SCN resistance and molecular marker background selection or straightforward line selection can be applied to implement the multiple SCN resistances identified in this study into favorable elite soybean cultivars. MAS directly improves selection gain, reducing breeding cost, and less “false” selected or “escapes” can happen as compared with solely field or bioassay selection. Potential linkage drags with undesirable traits should not cause any problems for cultivation or further improvement through breeding since the cultivar level has already been reached. This identification would be critical to achieving a better understanding of the molecular architecture of resistance to *H. glycines*. Our data suggest that there is a tremendous genetic potential available to improve soybean cultivars with resistance against *H. glycines* by using agronomically adapted and competitive *G. max* genetic breeding resources, as described in this study.

## Data availability statement

The original contributions presented in the study are included in the article/supplementary material. Further inquiries can be directed to the corresponding authors.

## Author contributions

YL and WL designed the experiments. MY supplied most of the cultivars. YL produced the data. YL drafted the manuscript with contributions from GK, HW, JL, BD, JW, and WL. All authors read and approved the submitted version.
